# Chronic rhinosinusitis with nasal polyps is characterized by dysbacteriosis of the nasal microbiota

**DOI:** 10.1038/s41598-018-26327-2

**Published:** 2018-05-21

**Authors:** Thanit Chalermwatanachai, Ramiro Vilchez-Vargas, Gabriele Holtappels, Tim Lacoere, Ruy Jáuregui, Frederiek-Maarten Kerckhof, Dietmar H. Pieper, Tom Van de Wiele, Mario Vaneechoutte, Thibaut Van Zele, Claus Bachert

**Affiliations:** 1 The Upper Airways Research Laboratory, Department of Oto-Rhino-Laryngology, Ghent University Hospital, Ghent, Belgium; 2 Department of Otolaryngology, Phramongkutklao Hospital and College of Medicine, Bangkok, Thailand; 3 Laboratory of Microbial Ecology and Technology, Ghent University, Ghent, Belgium; 4 Microbial Interactions and Processes (MINP) Research Group, Helmholtz Centre for Infection Research, Braunschweig, Germany; 5 Laboratory for Bacteriology Research, Faculty of Medicine & Health Sciences, Ghent University, Ghent, Belgium; 6 Division of ENT Diseases, Clintec, Karolinska Institute, Stockholm, Sweden

## Abstract

Chronic rhinosinusitis with nasal polyp (CRSwNP) patients are often characterized by asthma comorbidity and a type-2 inflammation of the sinonasal mucosa. The mucosal microbiota has been suggested to be implicated in the persistence of inflammation, but associations have not been well defined. To compare the bacterial communities of healthy subjects with CRSwNP patients, we collected nasal swabs from 17 healthy subjects, 21 CRSwNP patients without asthma (CRSwNP−A), and 20 CRSwNP patients with co-morbid asthma (CRSwNP+A). We analysed the microbiota using high-throughput sequencing of the bacterial 16S rRNA. Bacterial communities were different between the three groups. *Haemophilus influenzae* was significantly enriched in CRSwNP patients, *Propionibacterium acnes* in the healthy group; *Staphylococcus aureus* was abundant in the CRSwNP−A group, even though present in 57% of patients. *Escherichia coli* was found in high amounts in CRSwNP+A patients. Nasal tissues of CRSwNP+A patients expressed significantly higher concentrations of IgE, SE-IgE, and IL-5 compared to those of CRSwNP−A patients. Co-cultivation demonstrated that *P. acnes* growth was inhibited by *H. influenzae, E. coli* and *S. aureus*. The nasal microbiota of healthy subjects are different from those of CRSwNP−A and CRSwNP+A patients. However, the most abundant species in healthy status could not inhibit those in CRSwNP disease.

## Introduction

Chronic rhinosinusitis with nasal polyps (CRSwNP) is defined as a subgroup of chronic rhinosinusitis (CRS) that is characterized by the presence of fleshy swellings (nasal polyps) that develop in the lining of the nose and paranasal sinuses^1^. Nasal polyps (NP) are believed to arise in the nasal mucosa because of chronic inflammation. In central Europe, CRSwNP is mostly characterized by a moderate to severe T helper type 2 (Th2)-mediated inflammation with hypereosinophilia and increased IgE concentrations. CRSwNP is difficult to treat and recurrences are frequent, despite medical treatment and surgical interventions. In addition, patients suffering from CRSwNP often have comorbid diseases, such as asthma and aspirin intolerance^2,3^.

The definitive mechanisms underlying the pathogenesis of CRS remain poorly elucidated^4^. Microbial involvement have been considered to be one of the mechanisms contributing to the inflammation in case of CRS^5,6^. Whereas in some reports the characteristics of sinus microbiota in CRS were similar to those of control groups^7–9^, other analyses, making use of CRS phenotypes or endotypes, demonstrated different compositions of resident sinus bacterial communities^6,10^. Our group has previously demonstrated that *Staphylococcus aureus* is able to drive Th2 type inflammation and that it is associated with Th2-biased CRSwNP^5,6,11^. We have also reported that the expression of IL-5 and of IgE against *S. aureus* superantigens (SE-IgE) within polyp tissue is associated with comorbid asthma^12,13^. Furthermore, because the levels of IL-5 and SE-IgE were significantly increased in recurrent versus non-recurrent CRSwNP^14^ and because several studies suggest that alterations in the airway microbiota may be associated with inflammatory processes^6,7,10,15–17^. We reasoned that a study with well-defined subgroups of CRS patients could possibly identify different microbiota, which might be related to specific pathological or immunological characteristics of the inflammation.

Here, we hypothesized that heterogeneousness of CRSwNP with regard to asthma comorbidity could be associated with the presence of compositionally distinct sinus microbiota which affect host immune responses. To validate this hypothesis, we collected nasal swabs to obtain a suitable biological sample from the middle meatus, the common location of nasal polyps^18^, of CRSwNP patients without co-morbid asthma and compared their sinus microbiota to that of patients with CRSwNP with co-morbid asthma and to that of healthy control subjects, using 16S ribosomal RNA-gene (16S rRNA gene) high throughput sequencing. Furthermore, we studied the *in vitro* inhibitory and/or stimulatory effects of isolated strains of different species on each other, specifically to understand to what extent the species that are most frequently present in controls may protect the mucosa against germs most frequent in patients with CRSwNP.

## Materials and Methods

### Study Design and Population

We evaluated nasal microbiota from non-asthmatic CRSwNP patients, CRSwNP patients with co-morbid asthma, and healthy control subjects. Diagnosis of CRSwNP was made according to the European Position Paper on Rhinosinusitis and Nasal Polyps^19^. Diagnosis of asthma was confirmed by a pulmonologist. The atopic status was evaluated by skin prick tests to common inhalant allergens. The control group consisted of healthy volunteers and patients who were free from rhinosinusitis, asthma, and atopy. Patients less than 18 years of age, subjects with cystic fibrosis, known or suspected immunodeficiency or autoimmune disease, and/or suspected of the use of systemic antibiotics or oral steroids in the last 3 months before sample collection, were excluded from this study.

### Ethics statement

This study was approved by the ethics committee of the University of Ghent, Belgium and assigned number B670201422215. All participants provided written informed consent prior to their participation in the project. All experiments were performed in accordance with relevant guidelines and regulations.

### Sample Collection

Specimens were collected at the Ghent University Hospital, Belgium. Swab specimens for DNA extraction were obtained with eSwab (COPAN, Brescia, Italy). Swabs were endoscopically guided to the middle meatus region and rotated at least 3 times. In addition, nasal tissue samples were obtained from the patients during endonasal sinus surgery. All samples were immediately transported to the laboratory and snap frozen in liquid nitrogen and stored at −80 °C until further analysis.

### Measurement of Cytokines in Nasal Tissue Samples

Snap-frozen tissue specimens were weighed and suspended in a ratio 0.1 g of tissue per 1 mL of 0.9% NaCl solution with a complete protease inhibitor cocktail (Roche, Mannheim, Germany). To prepare soluble protein fractions, frozen tissues were mechanically pulverized using a Tissue Lyser LT (Qiagen, Hilden, Germany) at 50 oscillations per second for two minutes in prechilled Eppendorf tubes. Homogenized tissues were centrifuged at 1,800 × g for ten minutes at 4 °C, and the supernatants were collected. Total IgE, eosinophil cationic protein (ECP) and specific IgE to staphylococcal enterotoxins (SE-IgE) were measured using the UniCAP system (Thermo Fisher Scientific, Phadia, Uppsala, Sweden). Tumor necrosis factor-alpha (TNFα), interleukin (IL)-5 and IL-17 were quantified using the Bio-Plex 200 System (Bio-Rad, Temse, Belgium).

### DNA Extraction and Bioinformatic Analysis

DNA was extracted from the whole swab using the FastDNA Spin kit (MP Biomedicals, Solon, Ohio) according to the manufacturer’s instructions. Total DNA concentration was measured with a NanoDrop ND-1000 spectrophotometer (Isogen Life Sciences, IJsselstein, The Netherlands). The quality of the extracted DNA was evaluated on a 1% agarose gel. Libraries were prepared as previously described^20^, using the primers 27F and 338R^20^ for the V1-V2 region of the 16S rRNA gene. Libraries were sequenced on a MiSeq (Illumina, Hayward, California). Each sequence was identified using the SILVA database^21^. Definition of operational taxonomic units (OTUs) and data-set quality filter was performed as previously described^20^. After quality filtering, the dataset was then filtered to consider only those OTUs sequences that were present in at least one sample at a relative abundance of >0.1% or that were present in all samples at a relative abundance of >0.001%. All samples were randomly re-sampled to equal the smallest read size of 6,682 reads, using the PhyloSeq package^22^ from the free software R package for statistical computing and graphics^23^. All the reads were grouped into 942 sequences. All sequences were assigned a taxonomic affiliation (phylotype) based on the naive Bayesian classification (RDP classifier)^24^. A genus name was assigned to a sequences when only 16S rRNA gene fragments of previously described isolates belonging to that genus and of 16S rRNA gene fragments originating from uncultured representatives of that genus showed ≤2 mismatches. Species assignments were performed using the Basic Local Alignment Search Tool (BLAST). Assignation of a name demanded at least 99% sequence identity over 95% of sequence length^25^.

### Statistical Analysis

Statistical analysis was performed using GraphPad Prism version 6.00 for Mac OS X (GraphPad Software, La Jolla, CA: www.graphpad.com). The categorical outcomes (presence/absence of condition) were expressed as frequencies or percentages and were analyzed using the Chi-square test. The interval data (age, level of cytokines) were tested for distribution using the Shapiro-Wilk normality test. Assessment of the significance of intergroup correlation was performed using the parametric test for Gaussian distributed data and a non-parametric method for non-normally distributed data. The ecological profiles were analyzed using the PAST3 program^26^. All Principal Coordinate Analyses (PCoA) were based on a Bray-Curtis similarity index, which operated at the phylotype level. To test for community compositional differences, permutation multivariate analysis of variance (PERMANOVA) was employed. Similarity Percentage (SIMPER) analysis was used to determine the contribution of each species to the observed dissimilarity between samples and to identify the species that are most important in creating the observed pattern. In each group, the 20 bacterial species with the highest relative abundance were selected. This resulted in 22 different bacterial species for further analyses. The difference of relative abundance was calculated using a nonparametric Kruskal-Wallis test and corrections of significance for between-group comparisons were calculated using Dunn’s test. The Spearman’s rank correlation was employed to assess the statistical correlation between cytokine and bacterial relative abundance. The statistical significance level was determined as *p* < 0.05.

### Bacterial Cultivation and Interaction Assays

An agar diffusion test (Kirby–Bauer method) was employed to determine *in-vitro* interaction between bacterial strains. The bacterial strains were selected based on metagenomic and clinical data. In a separate group of patients (10 healthy control subjects, 10 CRSwNP−A patients, and 10 CRSwNP+A patients) we found that the majority of *S. aureus* strains isolated from CRSwNP patients expressed accessory gene regulator (*agr*) group I and *agr* group II. Reference strains expressing *agr* group I and II were therefore used in this assay. *Staphylococcus aureus* (gfp RN6390 and ATCC 29213), *Escherichia coli* (O157:H7 strain), *Haemophilus influenzae* (ATCC 49144), *Corynebacterium pseudodiphtheriticum* (CIP 103420T), and *Propionibacterium acnes* (ATCC 6919) were selected for the experiment. Chocolate agar plate inoculation and incubation at 37 °C with 5% CO_2_ was determined to be the culture condition that enabled proficient growth of all bacterial strains. Bacterial colonies were harvested, resuspended in 0.9% NaCl to a turbidity that was adjusted to a 0.5 McFarland standard. To test for evidence of cooperative or competitive interaction of bacteria, twelve culture plates were used, of which six were smeared uniformly with 0.5 McFarland suspensions of each of the six test strains to obtain confluent growth (inoculated plate) and the other six were not inoculated (control plates). After left to dry for 5 minutes, 10 µl of 0.5 McFarland standard suspensions of all six test strains were spotted on all culture plates. Agar plates were inspected at day 1, 2, 3, 5 and 7 for bacterial growth on the spots and also around the spots for the inoculated plates. The amount of growth of the spotted strains on the inoculated plates was compared to that on the control plates. Criteria for interpretation were established based on ecological interactions^27,28^. The nine possible interpretations of the interactions, ranging from mutually beneficial through neutral to spiteful interactions, are outlined in Table S1.

## Results

### Characteristics of The Patients

**Figure 1:**
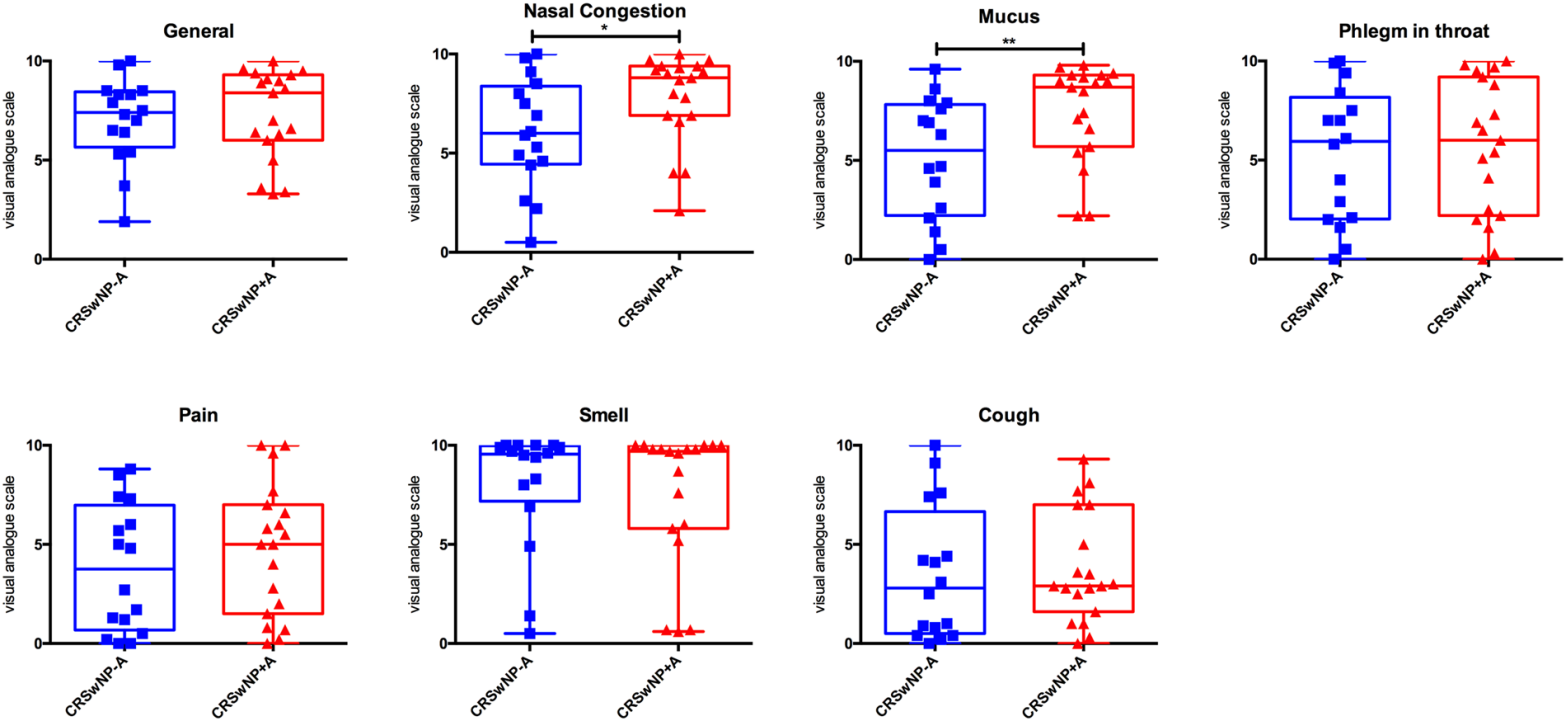
Visual analogue scale. The figure shows the symptom profile and symptom severity of the two patient groups. Nasal congestion and mucus scales were significantly higher in asthmatic patients compared to non-asthmatic patients. Statistical analysis was performed using Mann-Whitney test. *p < 0.05; **0.001 < *p* < 0.01.

Fifty-eight adults [17 control subjects, 21 CRSwNP patients without asthma (CRSwNP−A), and 20 CRSwNP patients with co-morbid asthma (CRSwNP+A)] were enrolled in the study. Table 1 illustrates clinical characteristics of the participants. No significant differences with regard to age or gender were found among the three groups. Atopic status and aspirin intolerance were significantly more prevalent in the CRSwNP+A group, as expected. According to severity of disease based on visual analogue scales for symptoms (VAS), CRSwNP+A patients had remarkably more trouble with nasal congestion and mucus than CRSwNP−A patients (Fig. 1).

**Table 1:** Characteristics of control and patient groups.

	Control	CRSwNP−A	CRSwNP+A	Statistical analysis*
Number of subjects	17	21	20	
Mean age (yr)	44.94	47.52	45.75	*p* = 0.8688
Male/Female	9/8	10/11	12/8	*p* = 0.7285
Atopy status positive (%)	0	19	80	*p* < 0.0001
Aspirin intolerance (%)	0	0	33.33	*p* = 0.0014

*Statistical test used. Kruskal-Wallis test for Age. Chi-square test for Gender, Atopy status, and Aspirin intolerance.

### Cytokine Patterns in The Nasal Tissue Samples

**Figure 2:**
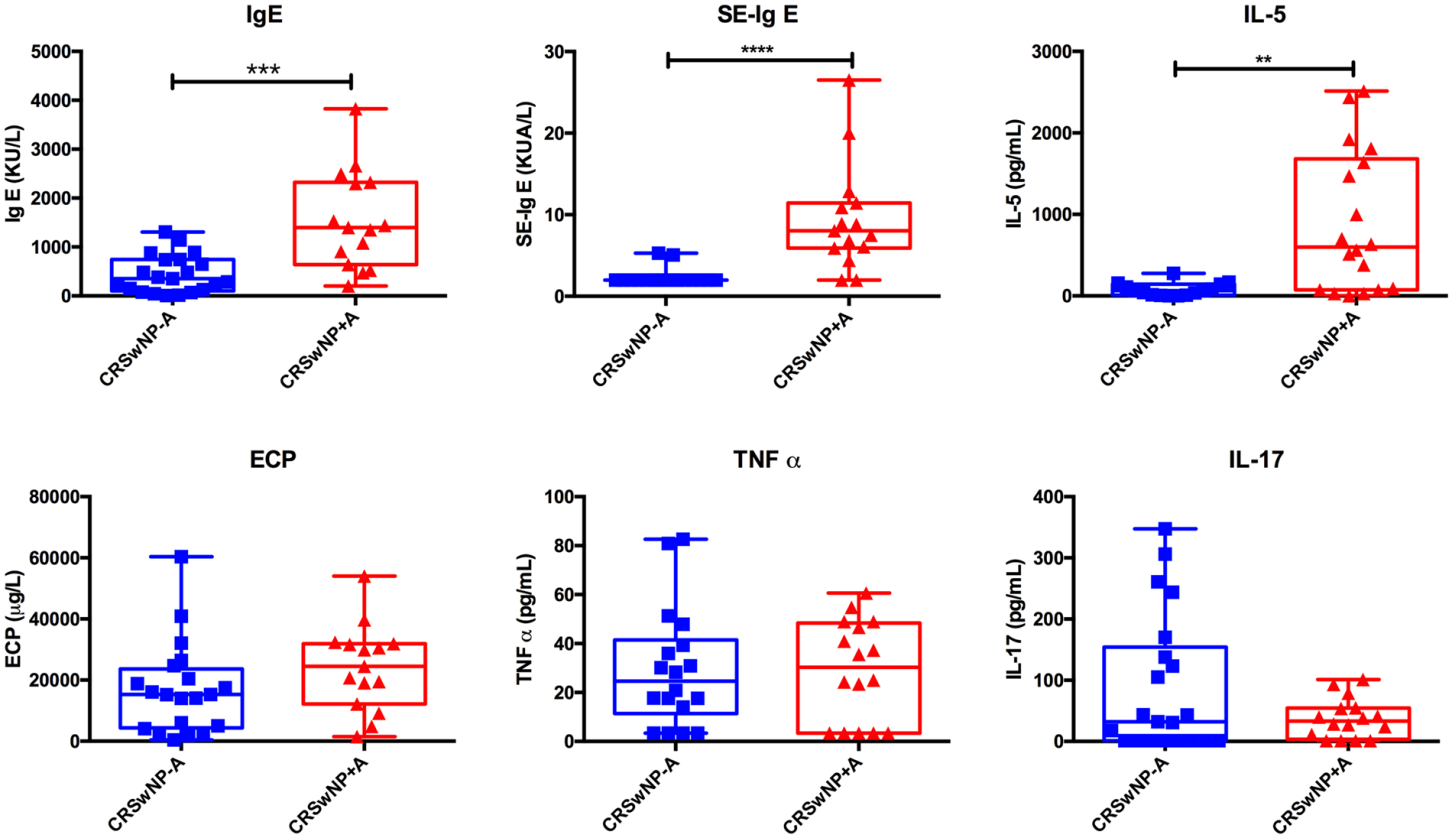
Cytokine expression in nasal tissues. The graphs show median, upper and lower quartiles. High IgE, SE-IgE and IL-5 concentrations characterize the inflammation in NP from asthmatic patients. Statistic results were calculated using the t-test for Gaussian-distributed data and the Mann-Whitney test for non-normal distribution. *p < 0.05; **p < 0.01; ***p < 0.001; ****p < 0.0001.

Nasal polyp (NP) disease in European patients is mostly characterized by an infiltration of eosinophils and expression of high concentrations of ECP. In this study, Th2 related mediators and cytokines (IgE, SE-IgE and IL-5) were significantly increased in NP from asthmatic patients compared with non-asthmatic subjects, as shown in Fig. 2. For example, median total IgE in the CRSwNP−A group was 440.4 kU/L (interquartile ranges IQRs: 101–742.5) compared to 1543 kU/L (IQRs: 640–2321) in the CRSwNP+A group (Table S2). The level of ECP, TNFα, and IL-17 showed no significant differences between groups. Furthermore, the SE-IgE positive group showed notable higher concentrations of IgE (median in the SE-Ig E positive subjects 1307 kU/L vs. 252 kU/L in SE-Ig E negative subjects, *p* < 0.0001), IL-5 (701 pg/mL vs. 77 pg/mL, *p* < 0.01), and ECP (29920 μg/L vs. 14045 μg/L, *p* < 0.01) when compared to the SE-IgE negative group.

### Microbial Diversity and Relative Abundances of Bacterial Species

**Figure 3:**
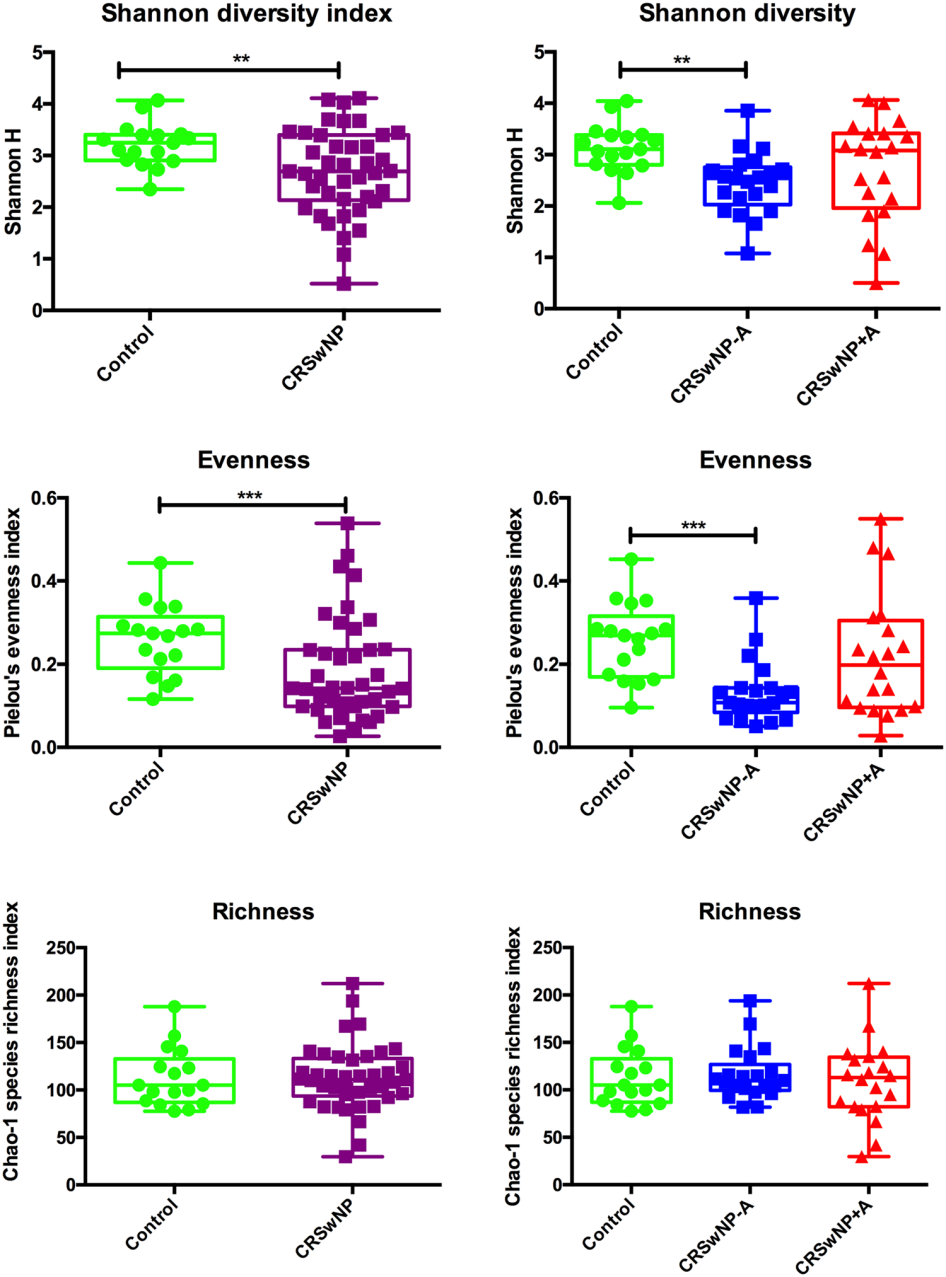
Biodiversity indices. The graphs compare diversity indices (Shannon diversity, richness, and evenness) between control subjects and patients with CRSwNP−A and CRSwNP+A. Statistical analysis was performed using Kruskal-Wallis and Dunn’s multiple comparisons test. **0.001 < *p* < 0.01; ***0.0001 < *p* < 0.001.

Bacterial DNA was detected in all samples. The number of phylotypes varied from 26 to 207 per sample with an average of 113 phylotypes in the control group, 116 phylotypes in the CRSwNP−A group, and 109 phylotypes in the CRSwNP+A group. Regarding the total number of reads of 16S rRNA gene, there was no statistically significant difference among the three groups, indicating that control subjects and CRSwNP patients had about the same total bacterial load. However, the sinonasal microbiota of CRSwNP as a group, regardless of co-morbid asthma, showed significantly decreased bacterial diversity (Shannon H index) and evenness (Pielou’s evenness index), when compared with the control group. Especially, the CRSwNP−A group exhibited significantly reduced bacterial evenness and Shannon’s diversity when compared with the control group. No significant differences were found with regard to Chao-1 species richness indexes in intergroup comparisons (Fig. 3).

**Figure 4:**
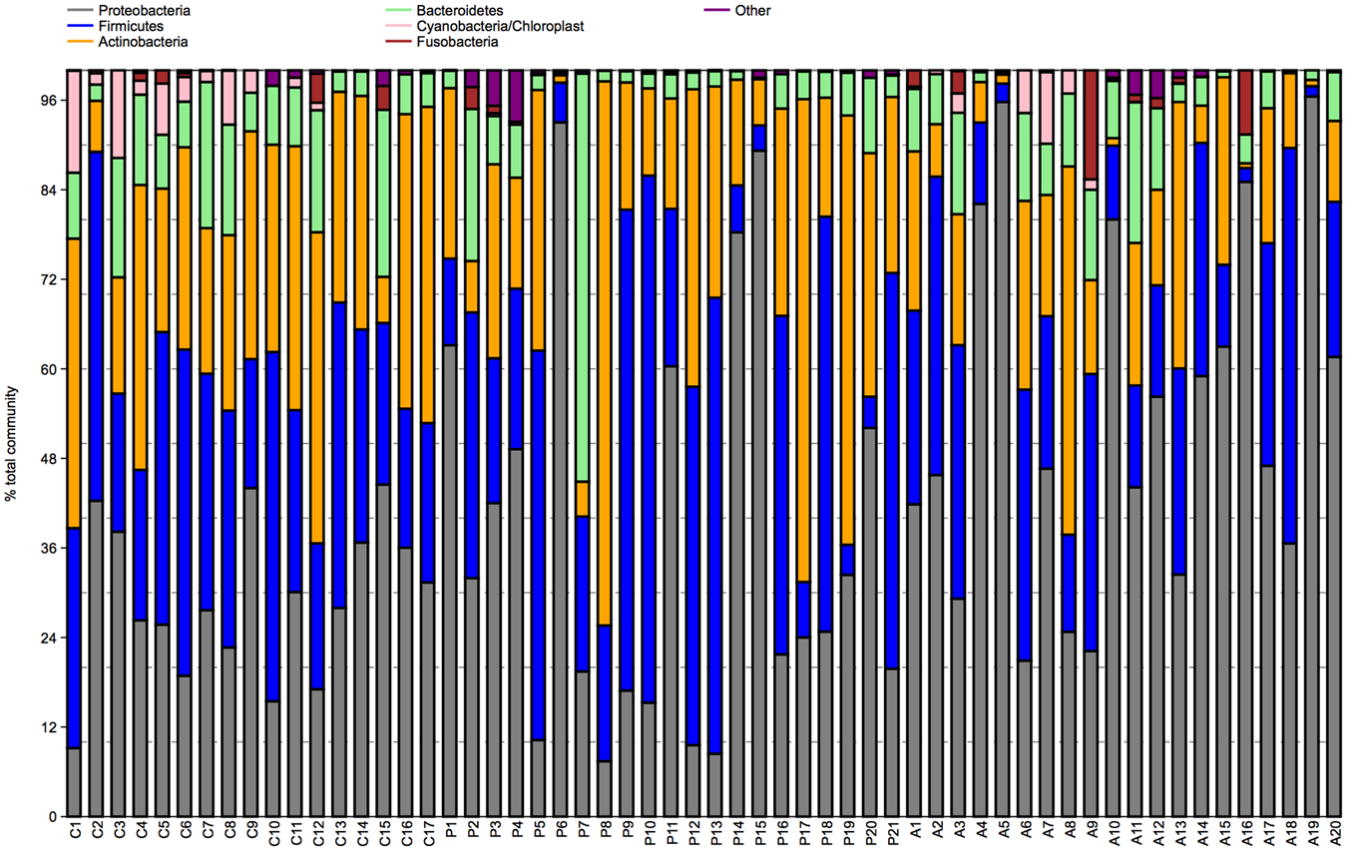
Bacterial community composition in each subject at the phylum level. C: control subject, P: CRSwNP−A subject, A: CRSwNP+A subject.

**Figure 5:**
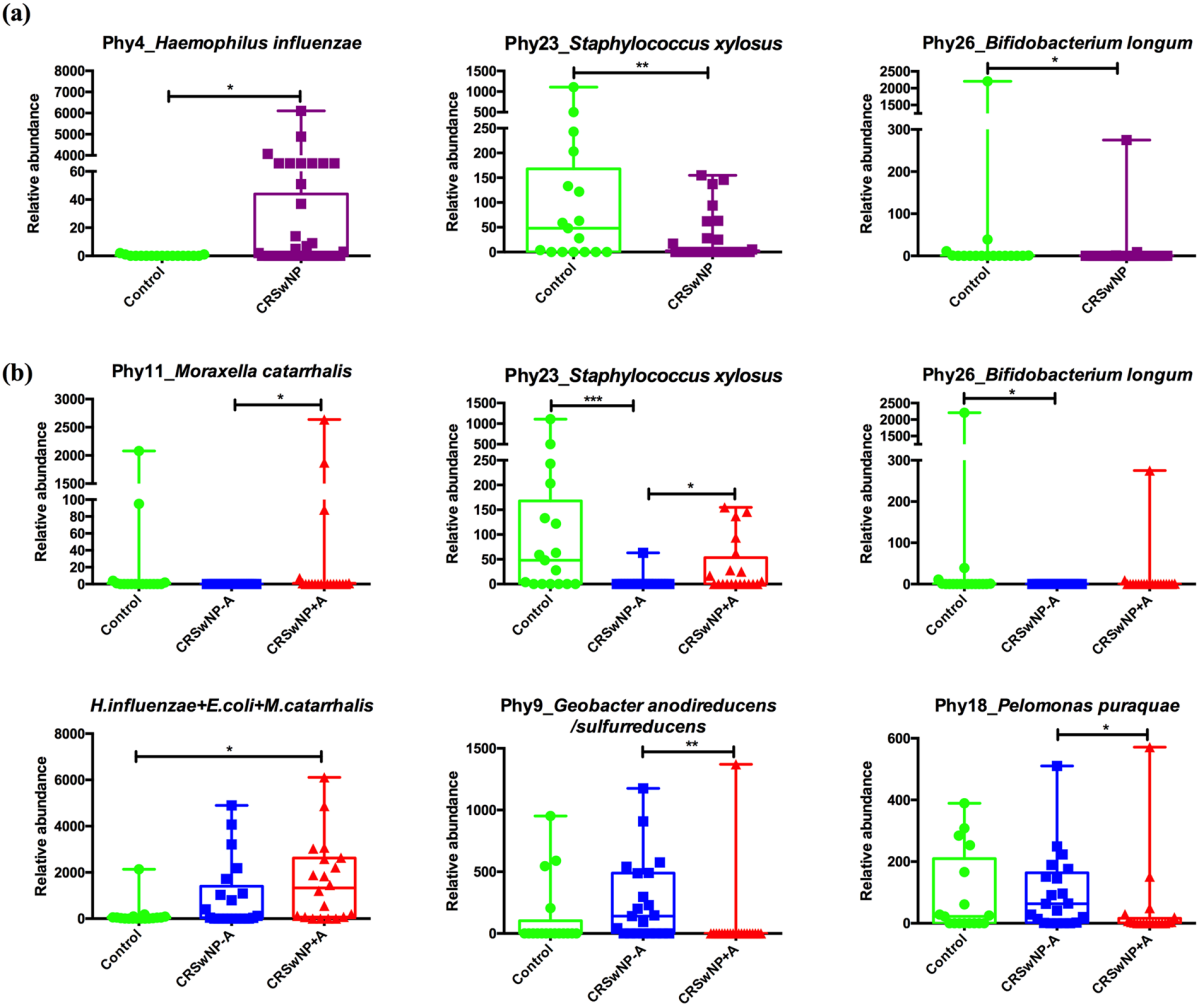
Bacterial species that discriminate between groups (**a**) and among groups (**b**). The graphs show the relative abundance of discriminative species different between groups. Dots represent relative abundance in each sample. *p < 0.05, **p < 0.01, and ***p < 0.001.

The phylum-level structure of our samples is depicted on Fig. 4. With respect to health and disease status, Phylum Proteobacteria and genus *Haemophilus* were more abundance in CRSwNP disease than healthy control, while the average abundances of phylum Actinobacteria, Bacteroidetes, and genus *Bacteroides* were dominant in healthy control subjects when compared with CRSwNP cases. At the species level, *Haemophilus influenzae* was significantly more abundant in CRSwNP patients than in control subjects. *Corynebacterium pseudodiphtheriticum* was more prevalent in diseases when compared with controls. *Staphylococcus xylosus* and *Bifidobacterium longum* were more prevalent and abundant in the control group than in the CRSwNP group (Fig. 5a).

A principal coordinate analysis (PCoA) ordination depicted sinus bacterial beta diversity between samples (Fig. S1). One-way PERMANOVA with Bray-Curtis index detected statistically significant differences of bacterial communities in intergroup comparisons (Holm-Bonferroni sequential corrected *p*-values < 0.001). A post-hoc pairwise comparison revealed differences in microbial composition between control and CRSwNP+A groups (*p* < 0.01), control and CRSwNP−A groups (*p* < 0.01), and between CRSwNP patients (*p* < 0.03).

**Figure 6:**
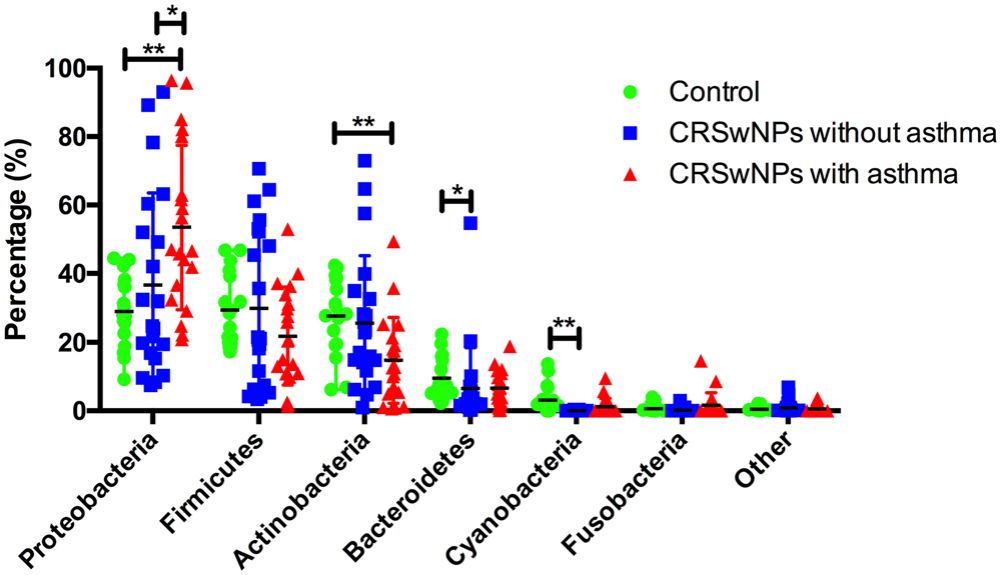
Phylum-level differences between groups. Dot graphs show relative abundance of bacterial phylafrom each sample. Kruskal-Wallis and Dunn’s multiple comparisons were employed for statistical analysis. *p < 0.05; **0.001 < *p* < 0.01.

Considering the bacterial composition at the phylum level (Fig. 6), Proteobacteria predominated in CRSwNP+A group compared with CRSwNP−A and control group, whereas Actinobacteria were more abundant in the control subjects than in patients with CRSwNP+A. The average abundances of Bacteroidetes were notably higher in control subjects when compared with CRSwNP−A patients. At the genus levels, CRSwNP−A patients carried significantly higher abundance of *Corynebacterium* (*p* = 0.04) genera (phylum Actinobacteria) and *Geobacter* (*p* < 0.01) genera (phylum Proteobacteria) when compared with CRSwNP+A patients (Fig. S2).

**Figure 7:**
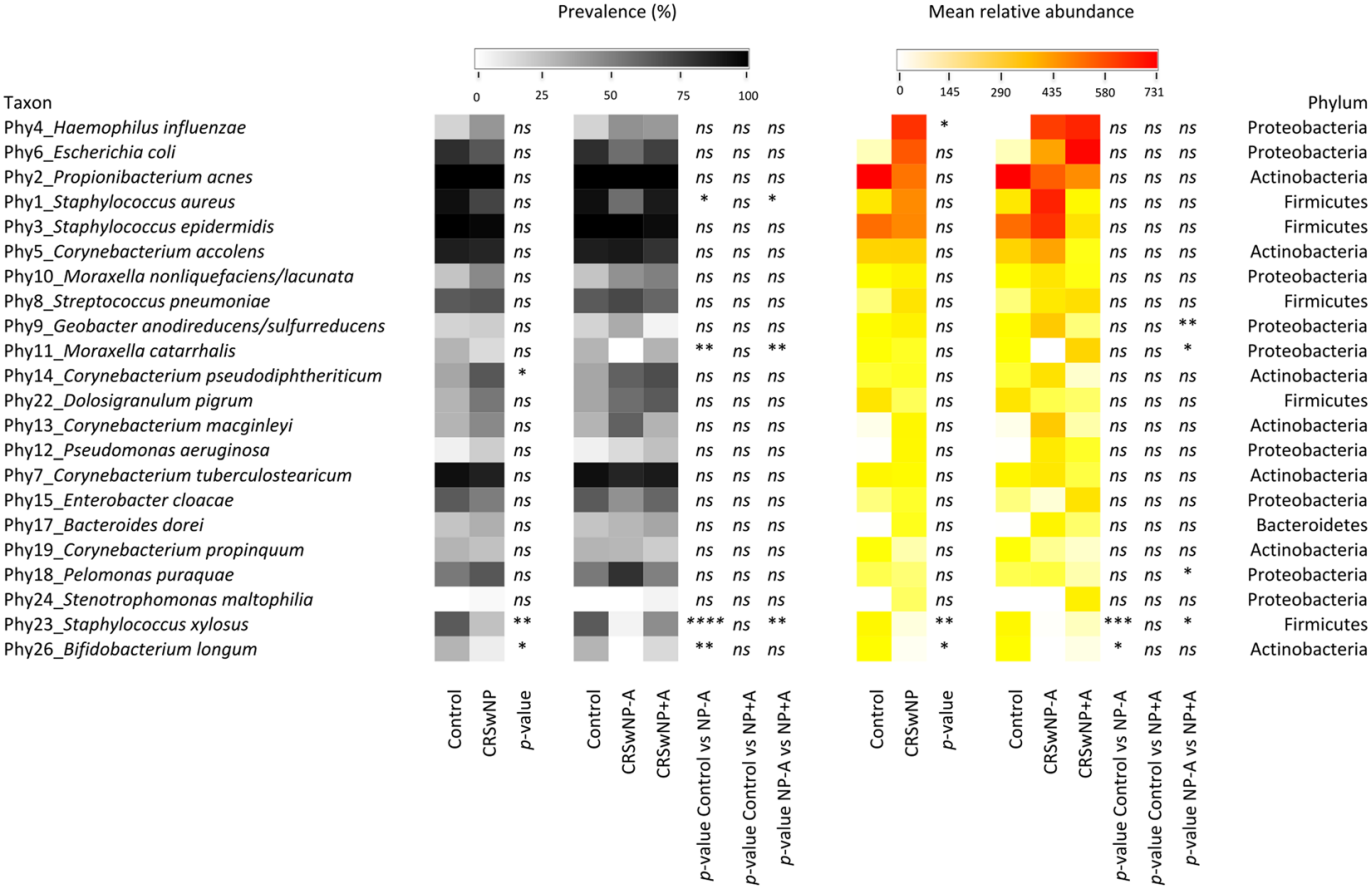
Prevalence and mean relative abundance of the most abundant bacterial species in our study. The heat maps in white-to-black and white-to-red depict the prevalence and relative abundance, respectively. The order was arranged based on the contribution to the observed dissimilarity. *ns:* no statistical significance (*p* ≥ 0.05); *p < 0.05; **p < 0.01; ***p < 0.001; ****p < 0.0001.

At the species level (Fig. 7), *Moraxella catarrhalis* was more prevalent and abundant in CRSwNP+A subjects, compared to CRSwNP−A subjects, whereas a relative abundance of *Geobacter anodireducens/sulfurrreducens* and *Pelomonas puraquae* was noted in CRSwNP−A subjects. All aforementioned species are member of the phylum Proteobacteria. The prevalence of *Moraxella catarrhalis, Staphylococcus aureus* and *Staphylococcus xylosus* was significantly lower in CRSwNP−A group when compared with the other groups (*p* < 0.05). The mean relative abundance of *Staphylococcus xylosus* was also notably lower in CRSwNP−A group when compared with the other groups. The prevalence and relative abundance of *Bifidobacterium longum* was higher in healthy group than CRSwNP−A group. For example, *Staphylococcus aureus* was prevalent in 94% of healthy control subjects, 57% of CRSwNP−A patients, and 90% of CRSwNP+A patients. The average relative abundance of *Staphylococcus aureus* was 185 in the healthy group, 654 in the CRSwNP−A group, and 145 in the CRSwNP+A group (Fig. S3). *Propionibacterium acnes* (phylum Actinobacteria) was the most abundant species in the control group, *Staphylococcus aureus* (phylum Firmicutes) in the CRSwNP−A group and *Escherichia coli* (phylum Proteobacteria) in the CRSwNP+A group.

### Correlations between Cytokine Levels and Relative Abundance of Bacteria

Comparison of cytokine levels with relative abundance of bacteria in the nasal polyps tissues revealed that ECP and IL-5 were negatively correlated with the phylum Actinobacteria (*p* < 0.05, *r* = −0.3652, and −0.3760 respectively). The phylum Bacteroidetes correlated positively with IL-5 (*p* < 0.02, *r* = 0.3969), but negatively with IL-17 (*p* < 0.01, *r* = −0.4632).

At the species level, total IgE and IL-5 were negatively correlated with *Geobacter anodireducens/sulfurreducens* and *Pelomonas puraquae*, IL-5 was negatively correlated with *Corynebacterium macginleyi*, and ECP was negatively correlated with *Corynebacterium accolens, C. macginleyi* and *Streptococcus pneumoniae*. In contrast, ECP and IL-5 were positively correlated with *E. coli*. *Staphylococcus aureus* and *Propionibacterium acnes* were not correlated with any cytokine. IL-17 and TNF-α were not correlated with any of the top 20 bacterial species (Table S3). However, no single correlation reached a correlation coefficient of higher than 0.6 (moderate correlation).

### Interspecies Interactions of Cultured Bacterial Species

When testing the possible interactions between six strains of five of the most frequently present species, we observed that growth of *C. pseudodiptheriticum* on the inoculated plate was enhanced by the presence of *S. aureus* RN6390 and *H. influenzae*, but retarded by *E. coli* and inhibited by *S. aureus* ATCC 29213. *P. acnes* appeared to be inhibitied by *S. aureus* (both strains), *E. coli* and *H. influenzae*. Furthermore, *H. influenzae* was inhibited by *S. aureus* ATCC 29213 and *E. coli*. Growth of *S. aureus* RN6390 was inhibited by *E. coli*.

**Figure 8:**
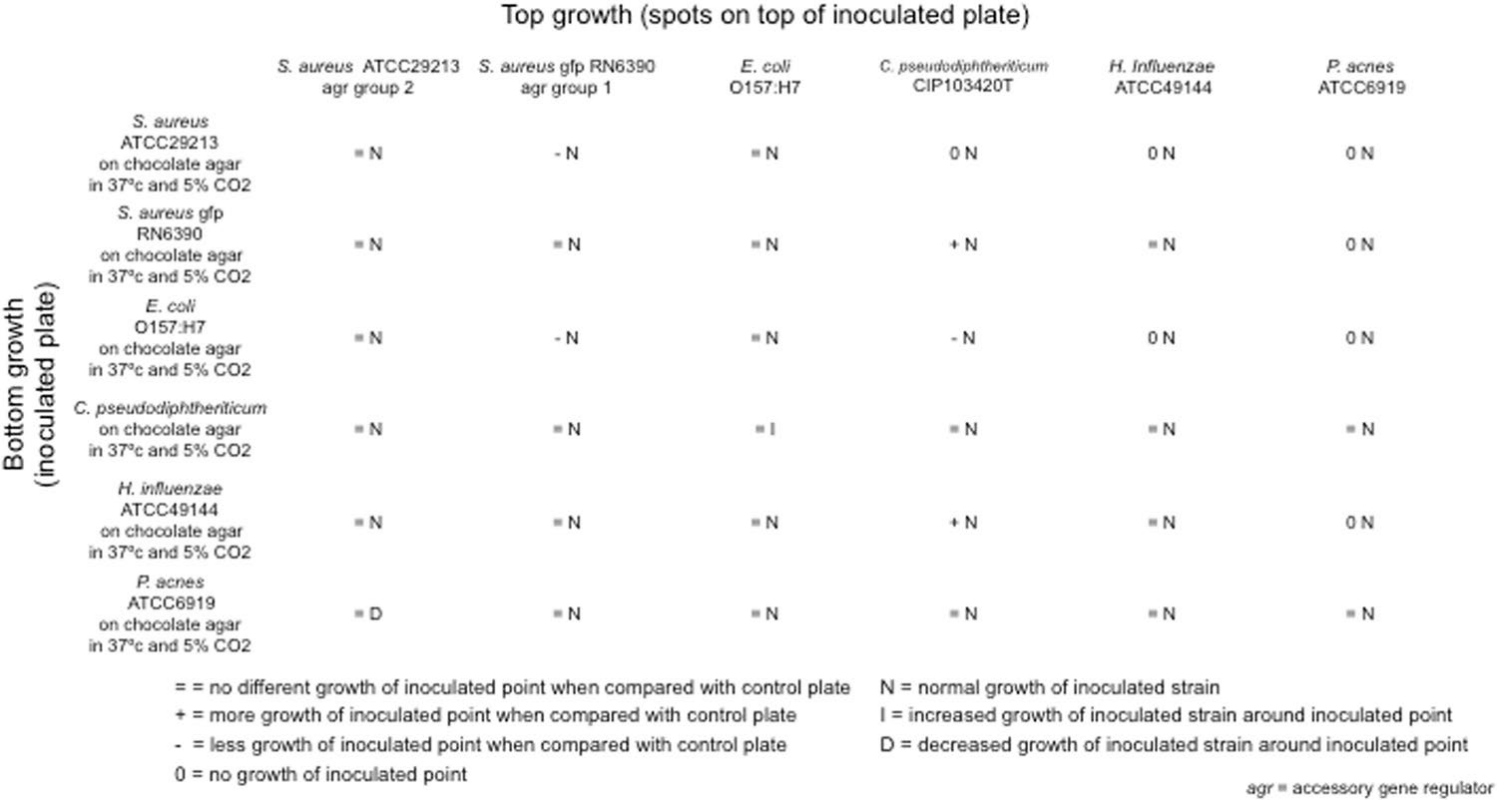
Mutual inhibition and growth promotion between different bacterial species that are most abundant in controls and patient groups.

**Figure 9:**
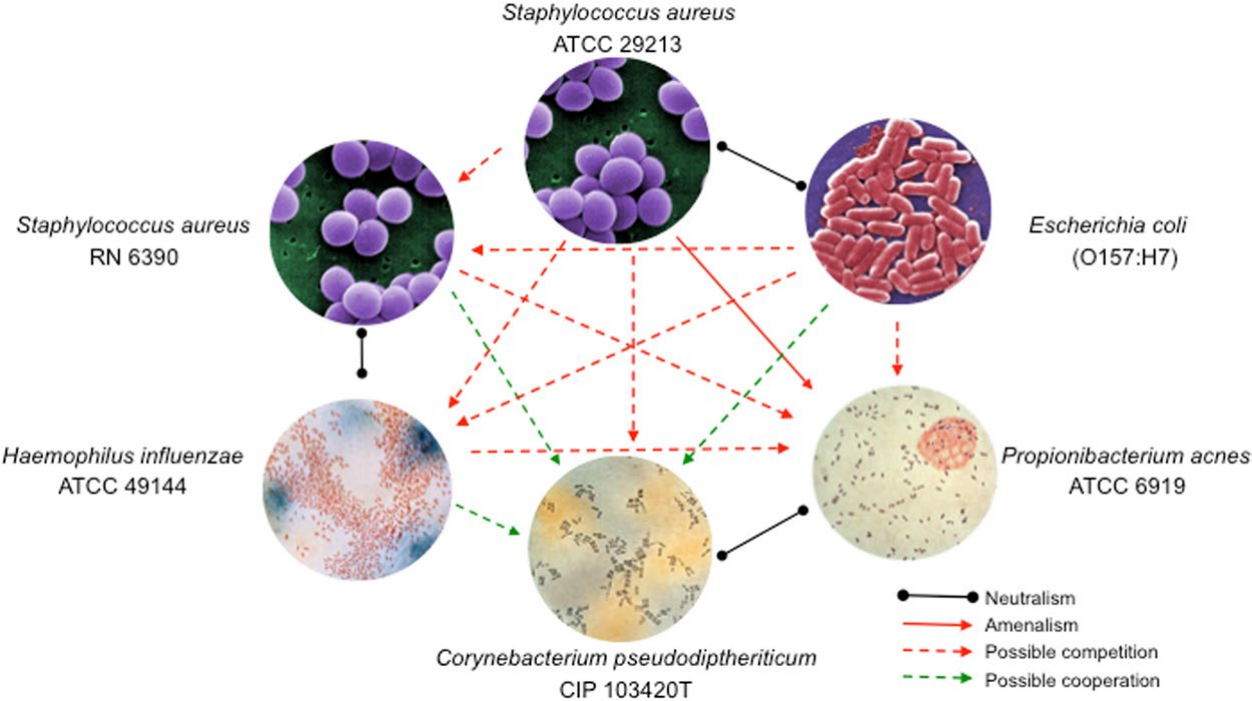
Bacterial *in vitro* interactions. Red lines represent a negative relationship, green lines represent a positive relationship, and black lines represent a neutral relationship. All bacterial pictures are credited to Centers for Disease Control and Prevention (CDC). The bacterial pictures were taken from Public Health Image Library (https://phil.cdc.gov/phil/home.asp). *C. pseudodiptheriticum* image ID#: 2126. *E. coli* image ID#: 10068 photo credit: Janice Harney Carr *H. influenzae* image ID#: 1947 *P. acnes* image ID#: 2122 *S. aureus* image ID#: 11157 photo credit: Janice Harney Carr.

With respect to growth pattern of the strains used to initially inoculate the plates, *C. pseudodiptheriticum* swiftly flourished in the proximity of the *E. coli* inoculation point, whereas growth of *P. acnes* was inhibited around the inoculation point of *S. aureus* ATCC 29213. Neither growth inhibition nor growth promotion was noticed between *C. pseudodiptheriticum and P. acnes*, and between *S. aureus* ATCC 29213 and *E. coli*. Figure 8 outlines the observed bacterial growth patterns. Interspecies interplay on the basis of the available information from our *in-vitro* experiments is depicted in Fig. 9.

## Discussion

The nasal bacterial community has been suggested to play a role in the development and severity of CRSwNP disease^29,30^. The aim of this study was to establish whether and to what extent there might be differences in the nasal microbiota of healthy control subjects, compared to those of non-asthmatic and asthmatic CRSwNP patients. Subgrouping of the patients into endotypes was based on our previous studies^3,6^. Indeed, CRSwNP disease can be further classified on the basis of distinct biomarkers (IgE, SE-IgE, and IL-5) that are increased in nasal polyps tissue when there is comorbidity (asthma). Different microbiota might represent different functional communities and invoke an immune response ranging from homeostatic to detrimental inflammatory effects, as such explaining the differences between the different endotypes.

### Aberrant bacterial community in CRSwNP disease

This study identifies that the relative abundance of *H. influenzae* is remarkably higher in CRSwNP cases compared to normal subjects. Combining the experimental data from mice^31^, the epidemiological data from humans^32^, and our observation of an association of *H. influenzae* with CRSwNP, we hypothesize that *H. influenzae* can initiate and drive inflammatory responses to develop nasal polyposis and therefore it may be hypothesized that elimination of *H. influenzae* in an acute phase might reduce incidence of NP cases. It might further be assumed that success or failure of the antibiotic treatment of the acute phase infection might drive an ongoing inflammation into resolution or NP formation, respectively.

In addition, the bacterial community in CRSwNP+A patients showed dominance of the phylum Proteobacteria, caused by unexpectedly high numbers of *E. coli* (*Enterobacteriaceae*), although not significantly different from the other two groups, whereas *S. aureus* was predominant in CRSwNP−A patients. Our findings concurred with a previous study that reported strong increase of *Enterobacteriaceae* in bronchial lavage of patients with asthma compared to control subjects^33^. When we combined the data of members of the Proteobacteria (i.e. *E. coli*, *H. influenzae*, and *M. catarrhalis*), we could establish a significant increase in relative abundance of these bacteria for patients with CRSwNP+A compared to the control group (Fig. 5b).

Proteobacteria might contribute to the pathogenesis of CRSwNP disease via eicosanoids and related mediators^34^. The abundance of *E. coli* in several CRSwNP+A patients and the positive correlation of *E. coli* with ECP and IL-5 suggest a role of *E. coli* in severity of type 2 inflammation in CRSwNP patients.

It is possible that *E. coli* impairs the epithelial barrier and cooperates with putative pathogens for initiation and amplification of type 2 inflammation in CRSwNP disease.

### Effect of microbial components on putative pathogens

In the current study, the average relative abundance of *S. aureus* in CRSwNP−A patients is higher than that in healthy control subjects, but this is not the case for CRSwNP+A patients. The presence of specific IgE to staphylococcal enterotoxins (SE-IgE) is significantly more frequent in the CRSwNP+A compared to the CRSwNP−A and control groups and correlates with disease severity. This observation supports the role of *S. aureus* and its immune proteome in the persistence of airway disease^35,36^. Apparently, our findings seem to indicate that the composition of the bacterial community may influence the *S. aureus* virulence. The presence of large numbers of Actinobacteria (e.g. *Corynebacterium spp*.) in the environment might shift *S. aureus* to benign colonization, whereas increased numbers of Proteobacteria (predominantly *E. coli*) in the milieu might drive *S. aureus* to exhibit pathogenic behavior. It may be conceivable that bacterial composition and interactions in the community culminate in a functional community that organizes commensal-pathogen interchange. Intraspecific variability cannot be ruled, whereby *S. aureus* strains present in healthy samples could differ from strains from samples from diseases patients. In one study, a strain of *S. aureus* inhibited other strains of the same species co-colonizing in nasal cavity^37^. Various hypotheses were proposed to determine the regulation of *S. aureus* virulence, of which the global control of accessory gene regulator (*agr*) is the most promising one^38^. We also identified strain-level variations of *S. aureus*, classified by *agr*, in an independent survey and *in-vitro* bacterial interaction experiments. *S. aureus* RN 6390 belonging to *agr* group I and *S. aureus* ATCC 29213 *agr* group II. *S. aureus agr* group I had a positive impact on *C. pseudodiptheriticum*, whereas *S. aureus agr* group II had a negative impact on *C. pseudodiptheriticum*. Moreover, *S. aureus agr* group II killed *P. acnes* in our experiments. A survey of *S. aureus agr* in higher numbers of patients should be conducted to confirm the possible importance of *agr* diversity of *S. aureus* in pathogenesis of CRS diseases.

*Corynebacterium* species have been implicated as competitors with *S. aureus* in the nasal niche^39,40^ or as tempering *S. aureus* virulence^41^. One research group observed that *C. pseudodiphtheriticum* was present more often in non-*S. aureus* carriers than that in *S. aureus* carriers^42^. This group confirmed a competitive interaction between *S. aureus* and *C. pseudodiphtheriticum*^42^, which is not supported by our data. We detected *C. pseudodiptheriticum* more frequently in the CRSwNP status. In addition, our *in-vitro* interactions rather indicated a positive cooperation between *C. pseudodiptheriticum* and putative pathogens, such as *E. coli*, *H. influenzae* and *S. aureus (agr* group I).

Although several studies have described a negative association between *S. aureus* and *S. epidermidis* in the nasal cavity^40,43^, our data did not show competition between *S. aureus* and *S. epidermidis*.

### Bacterial Associations with Inflammation in CRS

A study using only 7 samples from CRS patients and a not-validated mice model concluded that *Corynebacterium tuberculostearicum* was a pathogen of CRS^44^, while another study reported that CRS patients with enriched *C. tuberculostearicum* colonization in their nose, at the time of endoscopic sinus surgery, showed better surgical outcomes^10^. However, in our study neither a beneficial nor pathogenic role of *C. tuberculostearicum* can be concluded. We found that the distribution and relative abundance of *C. tuberculostearicum* was equivalent among groups. We also observed a negative correlation between eosinophilic activity in the tissue (i.e., ECP and IL-5) and other members of genus *Corynebacterium* (i.e., *C. macginleyi*, and *C. accolens*) that belong to phylum Actinobacteria. According to our previous study, the level of IL-5 is one of the prognostic factors in CRSwNP disease^14^. These results suggest that members of phylum Actinobacteria, i.e.,*Corynebacterium spp*. (e.g. *C. macginleyi*), are associated with a lower degree of eosinophilic inflammation in CRSwNP patients.

### Health-associated Microbes

Several bacterial phyla were dominant in healthy individuals. The potentially beneficial microbe in the current study was a member of the phylum Actinobacteria; we found that *Propionibacterium acnes* was the most abundant microorganism in the healthy condition. Normally, *P. acnes* is a commensal microbe of adult human skin^45^. *P. acnes*, which prefers a lipid-rich anaerobic environment, has been detected in sinonasal cavities of healthy adults^9,42,43,46^*. P. acnes* has been described to promote Th1 and Treg cells and relieves atopic dermatitis symptoms^47^, possibly by the production of short-chain fatty acids that are immunomodulating. Moreover, the principal metabolite of *P. acnes*, propionic acid, not only reduces inflammation by effects on macrophages^48^, but also inhibits *S. aureus* growth^49^. This argument nevertheless remained unsupported by our pilot study. The levels of propionic acid measured in the sinonasal environment did not reach the minimum inhibitory concentration of *S. aureus*. Furthermore, *P. acnes* did not prevent the growth of *S. aureus*, which in turn killed *P. acnes* to establish its own growth. These data do indicate that *P. acnes* is an commensal rather than a defender, although *in-vitro* data regarding to bacterial interactions cannot be straightforwardly transposed to the *in-vivo* situation. *P. acnes* probably possess other factors for its fitness in healthy subjects. Healthy hosts may provide nutrients for *P. acnes* existence. Health-associated microbes should be further investigated to identify a genuine probiotic for the treatment of CRS disease.

**Figure 10:**
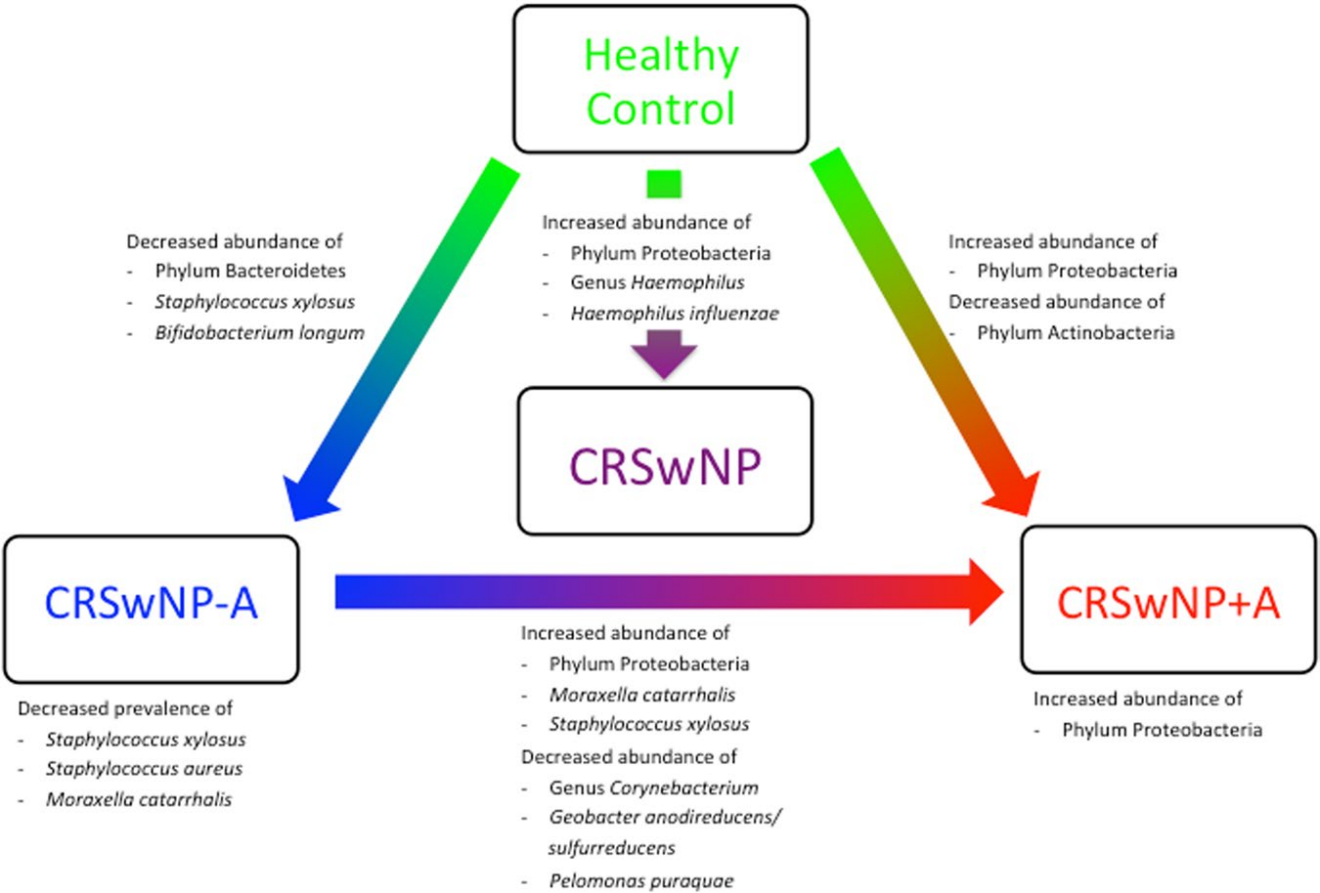
Characteristics of bacterial communities for the different groups.

This study provides a blueprint for identifying important microbial influencers of disease that provide information of structure and member of nasal microbiota linked to clinical phenotypes and endotypes such as subgroups of CRSwNP disease (Fig. 10). Our findings highlight the need for more functional studies of bacterial community to determine the role of microbiota in CRS pathogenesis.

### Limitations

Several limitations should be addressed. Although we excluded patients that used steroids systemically, all CRSwNP patients may have taken intranasal steroid until shortly before the biopsy. The effect of steroids on bacterial communities remains unclear, although nasal irrigation with budesonide did not change the proportional abundance of bacteria in CRSwNP patients^50^. We were concerned about the specificity of molecular techniques, even though it has been shown that the relative abundance of bacterial DNA had a positive correlation with colony-forming unit counts (CFUs)^30^. Comparing recovered DNA to live colony counting, it appeared that in diseased subjects, *Staphylococcus* DNA mostly came from living bacteria, whereas in healthy subjects, *Staphylococcus* DNA was mostly recovered from dead bacteria^51^. The method used here for microbiota may detect genetic material of non-viable microorganisms that distort the genuine microbial community composition. Moreover, one bacterial cell carries from 1 up to 15 copies 16S rRNA gene copies per genome^52^. The analysis of 16S rRNA can provide only the information of bacterial presence, but not metabolically active microorganism. The extraction and analysis of metagenomic mRNA (the metatranscriptome) or proteomic or metabolomic research should be further performed to assess the functional and metabolic diversity of microbial communities. Interplays between bacteria in our experiments are performed under *in-vitro* conditions. Although the tested strains were not directly isolated from the subjects studied, and relevant variations in the metabolites of the microbes may occur, the selected strains were chosen based on investigations in the same patient groups to best represent the situation in the human nose. We tested the interaction of only two species of bacteria. Interactions among the full spectrum of bacteria *in-situ* conditions may be different. The participation of the third, forth, and more species probably affects those bacterial behavior differently. A complementarily ecological theory would need to explain overarching interactions and patterns for the microbiota. Future research should explore functional parts of bacterial community and their impacts on the nasal mucosa. Investigations of bacterial communities and their impacts on sinonasal immune responses may pave the way for a better understanding of how the microbiota contribute to the pathogenesis of CRS.

## Conclusions

This study identifies a difference in nasal microbiota between healthy subjects and phenotypes of CRSwNP patients. Proteobacteria (such as *H. influenzae, E.coli, M. catarrhalis*) were associated with CRSwNP disease, especially with CRSwNP+A cases, whereas Actinobacteria (such as *P. acnes*, *Corynebacterium spp*.) were prevalent in the healthy status. However, *P. acnes*, a resident commensal bacterium in healthy subjects, does not play the protective role against *S. aureus* one could expect from its abundance. Furthermore, although *S. aureus* was found abundant in CRSwNP−A, but not in CRSwNP+A, the presence of SE-IgE in the latter group representing its immunologic fingerprint rather than *S. aureus* itself is associated with disease severity. The functions of microbiota and their products in health and airway disease should be further elucidated to determine the effective role of microbiota in the pathomechanism of CRS disease.

## Electronic supplementary material

Supplementary information

Dataset 1
