# Supplementary material for: Chronic rhinosinusitis with nasal polyps is characterized by dysbacteriosis of the nasal microbiota
Source: Sci Rep. 2018 May 21;8:7926. doi: 10.1038/s41598-018-26327-2 (PMC5962583; doi:10.1038/s41598-018-26327-2)
Supplement: Supplementary file 1 — Supplementary information [file 41598_2018_26327_MOESM1_ESM.docx]

Supplementary Information

Chronic rhinosinusitis with nasal polyps is characterized by dysbacteriosis of the nasal microbiota

Thanit Chalermwatanachai, Ramiro Vilchez-Vargas, Gabriele Holtappels, Tim Lacoere, Ruy Jáuregui, Frederiek-Maarten Kerckhof, Dietmar H. Pieper, Tom Van de Wiele, Mario Vaneechoutte, Thibaut Van Zele, Claus Bachert

Supplemental tables

Table S1. Criteria for interpretation of bacterial interaction.

Legend: >: more growth of inoculated strain on inoculated plate when compared to control plate, =: no difference in growth; <: less growth

| Bottom growth  (inoculated plate) | Top growth (spots on top of inoculated plate) | | |
| --- | --- | --- | --- |
|  | > | = | < |
| > | Mutualism | Possible cooperation | Selfishness |
| = | Commensalism | Neutralism | Amenalism |
| < | Altruism | Possible competition | Spite |

Table S2. Cytokine concentrations of homogenizing nasal tissue: CRSwNP-A versus CRSwNP+A

| Cytokine (unit) | CRSwNP-A | CRSwNP+A | *p*-value |
| --- | --- | --- | --- |
| Ig E (KU/L) | 440.4 (101-742.5) | 1543.0 (640-2321) | 0.0008 |
| SE-IgE (KUA/L) | 1.9 (1.9-1.9) | 8.0 (5.9-11.4) | <0.001 |
| IL-5 (pg/mL) | 63.1 (24.3-150.7) | 634.3 (73.6-1639) | 0.0018 |
| ECP (μg/L) | 15274 (4343-23654) | 24486 (10672-32109) | 0.0922 |
| TNF-α (pg/mL) | 25.1 (3.4-48.9) | 30.1 (15.8-50.4) | 0.7898 |
| IL-17 (pg/mL) | 32.0 (1.1-154) | 28.4 (1.1-54.7) | 0.5628 |

Values are expressed as median (interquartile ranges); differences between groups are determined using Mann-Whitney test.

Table S3. Significant correlations between cytokines and relative abundance of top 20 bacterial species in the sinus microbiota of CRSwNP patients

| Species | Immune marker: Coefficient (*p*-value) | | |
| --- | --- | --- | --- |
|  | Total IgE | ECP | IL-5 |
| *Bacteroides dorei* |  |  | 0.4472 (0.0062) |
| *Corynebacterium accolens* |  | -0.3880 (0.0213) |  |
| *Corynebacterium macginleyi* |  | -0.5658 (0.0004) | -0.4030 (0.0148) |
| *Enterobacter cloacae* |  | 0.3393 (0.0461) | 0.3377 (0.0439) |
| *Escherichia coli* |  | 0.4638 (0.0050) | 0.4150 (0.0118) |
| *Geobacter anodireducens/*  *G. sulfurreducens* | -0.4510 (0.0058) |  | -0.3704 (0.0262) |
| *Pelomonas puraquae* | -0.3563 (0.0330) |  | -0.3526 (0.0349) |
| *Streptococcus pneumoniae* |  | -0.3539 (0.0370) |  |

Legend: ECP: eosinophil cationic protein

Supplementary figures

Fig.S1. PCoA plot of Bray-Curtis distance between control subjects (green dot), CRSwNP-A patients (blue square) and CRSwNP+A patients (red triangle).


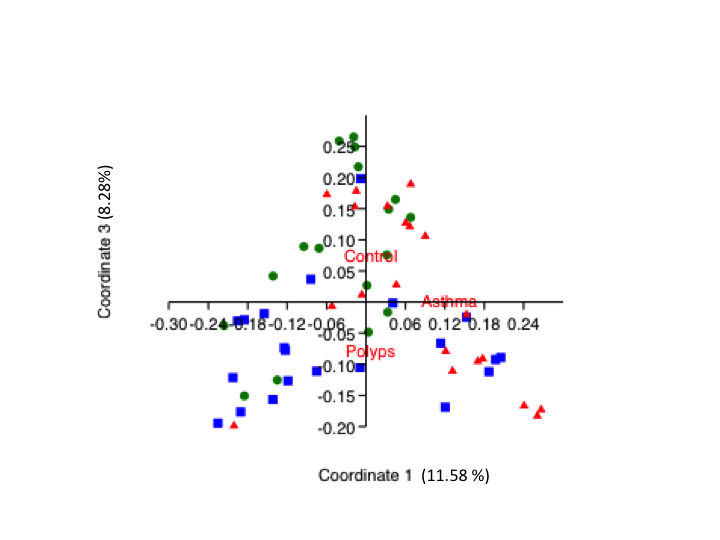


Fig.S2. Heatmap analysis of the most abundant bacterial genera. The heatmap shown in white-to-red represents relative abundance that discriminates between groups. The difference of abundance was calculated using a nonparametric Kruskal-Wallis test and corrections of significance for between-group comparisons were calculated using Dunn's test. Asterisks denote significant *p*-values.

Legend: **p* < 0.05, and ***p* < 0.01.

Fig S3 Result of the bacterial community in the study
